# Combined Endovascular Repair of a Giant Symptomatic Hepatic Aneurysm: A Case Report and Comprehensive Literature Review

**DOI:** 10.7759/cureus.62228

**Published:** 2024-06-12

**Authors:** Konstantinos Tigkiropoulos, Katerina Sidiropoulou, Manolis Abatzis-Papadopoulos, Dimitrios Karamanos, Ioannis Lazaridis, Nikolaos Saratzis

**Affiliations:** 1 1st Surgical Department, Division of Vascular Surgery, Papageorgiou General Hospital, Aristotle University, Thessaloniki, GRC

**Keywords:** covered stent, embolization, endovascular repair, giant aneurysm, hepatic artery aneurysm

## Abstract

Hepatic artery aneurysms (HAAs) are an uncommon vascular disease, which account for 20% of visceral artery aneurysms. The majority are usually asymptomatic and discovered accidentally during imaging control, but occasionally, they can present as acute abdominal pain, haemobilia, obstructive jaundice, or gastrointestinal bleeding due to aneurysm sac expansion or rupture with catastrophic consequences. We present the case of a 51-year-old male patient with a giant common HAA of 11.1 cm who was managed endovascularly. A combined endovascular approach was decided due to the anatomy of the aneurysm. Endovascular embolization with coils in the distal part of the aneurysm and deployment of a stent graft proximally to exclude inflow were used. At six months, the aneurysm size was regressed at 5 cm; however, seven months after the operation, the patient presented with pylorus perforation due to coil migration which was managed by coil removal, peripheral gastrectomy, and Roux-en-Y gastric bypass. We provide a narrative literature review regarding the endovascular repair of giant HAAs. The PubMed, Scopus, and Google Scholar databases were searched for articles up to January 2024. Thirty-eight studies (case reports, case series) were retrieved. The conclusion is that giant HAAs are a rare and severe condition in which their treatment can be challenging with unexpected adverse events. The literature review suggests that the endovascular approach whenever feasible is a safe and effective treatment option with low morbidity and mortality.

## Introduction

Splanchnic artery aneurysms are an uncommon but life-threatening vascular condition, with an incidence of 0.1-2% [1,2]. Hepatic artery aneurysms (HAAs) are the second most common splanchnic artery aneurysms. They represent 20% of all splanchnic aneurysms, and they have a 44% risk of rupture with high mortality rates [3]. The majority occur in the common hepatic artery and the proper hepatic artery, while men are more affected than women [4]. Giant HAAs are defined as aneurysms with a diameter >5 cm [5]. We present the case of a male patient with a giant symptomatic common HAA, who was treated with combined endovascular means, coil embolization, and stent graft deployment. A review of the current literature regarding endovascular repair of giant HAAs was additionally performed.

## Case presentation

**Figure 1:**
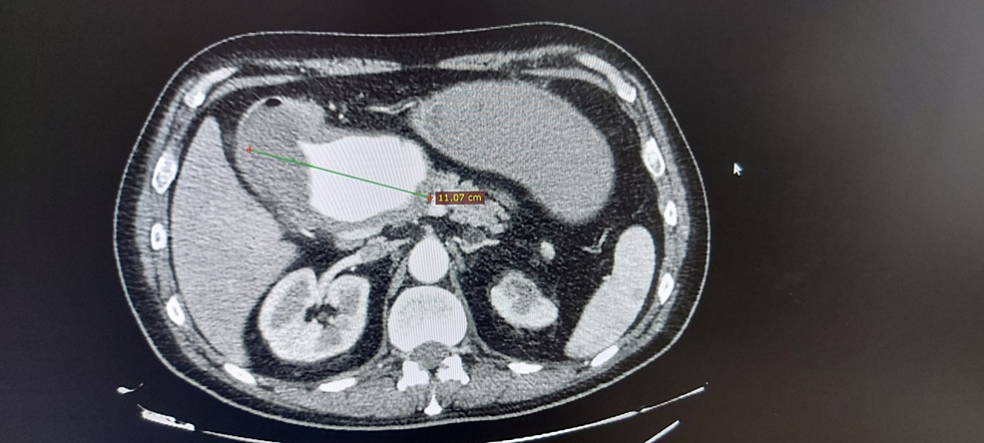
Contrast-enhanced CT axial view of the giant hepatic aneurysm. CT: computed tomography

**Figure 2:**
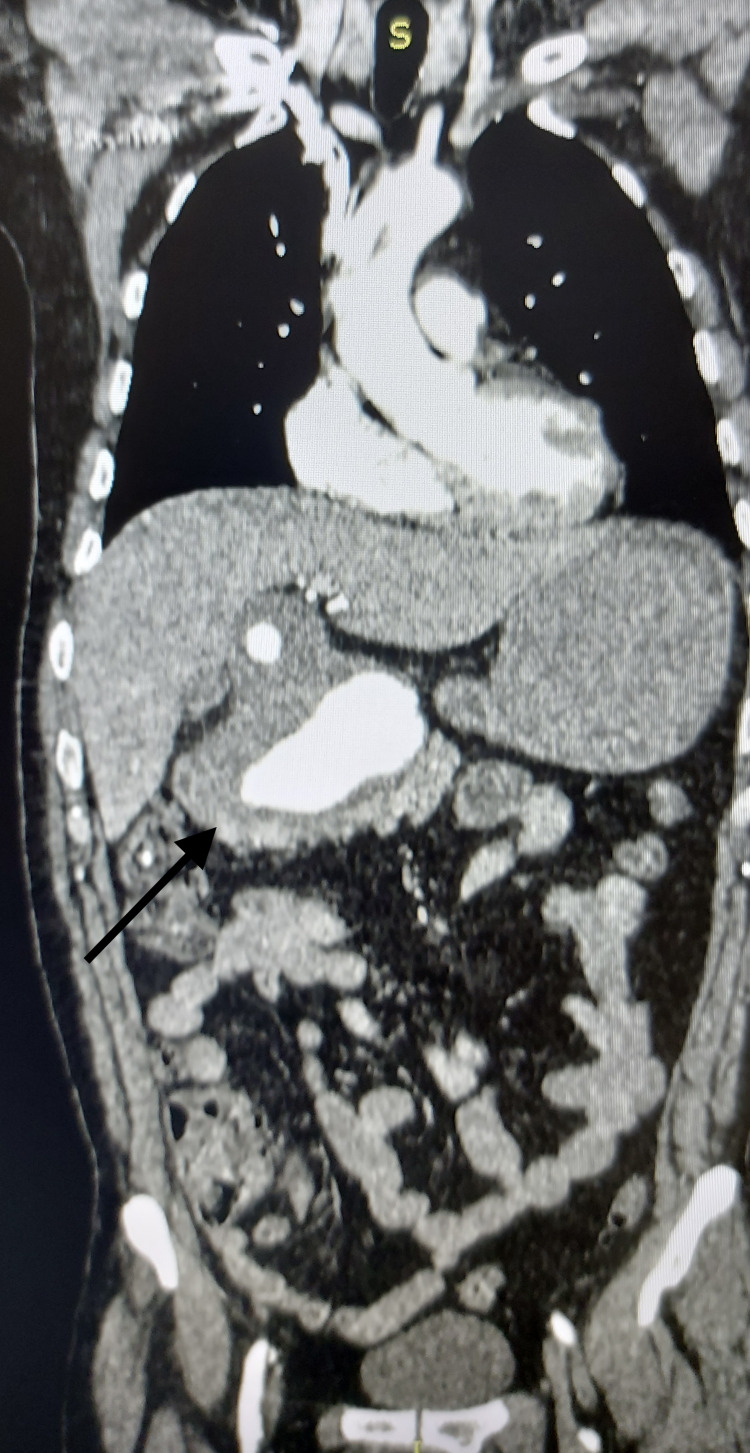
Contrast-enhanced CT coronal view of the giant hepatic artery (black arrow). CT: computed tomography

A 51-year-old Caucasian male patient was referred to our hospital from a secondary medical unit with abdominal and lower back pain. His medical history included arterial hypertension and multiple vertebral body fractures after a car accident 15 years ago. The patient did not refer alcohol consumption, smoking, and a recent infectious disease. A contrast-enhanced computed tomography (CT) was performed which revealed a giant common HAA of a maximum diameter of 11.1×6.8 cm with mural thrombus and no signs of rupture (Figure 1, Figure 2).

The aneurysm was expanding from the celiac artery bifurcation to the liver hilum. The portal venous system was intact without the presence of thrombosis due to possible compression from the aneurysm. The gastroduodenal artery and the proper hepatic artery and its bifurcation were not identifiable at CT. Due to the anatomy of the aneurysm, an endovascular approach was deemed feasible. The patient was informed of his treatment options (open and endovascular) and possible adverse events, and endovascular repair was chosen. A written informed consent was obtained from the patient.

**Figure 3:**
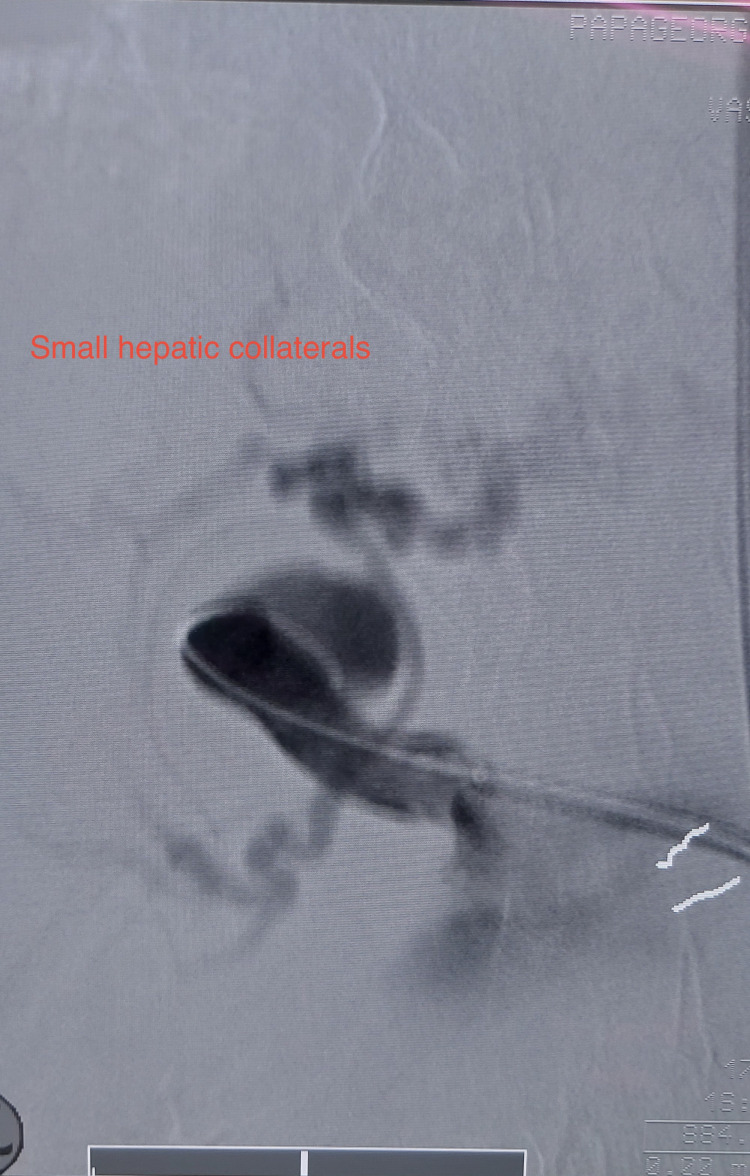
Intraoperative angiography revealed the presence of small collaterals in the liver hilum and the absence of right and left hepatic arteries.

Under general anesthesia, the left arm and abdomen were prepped and draped. Vascular access was gained by the surgical cutdown of the left brachial artery. After systemic heparinization (75 units/kg), a 5-Fr introducer sheath was introduced, and a 0.035-inch guidewire (Radifocus™, Terumo, Tokyo, Japan) was advanced to the abdominal aorta with a RIM catheter (Boston Scientific, Marlborough, Massachusetts, United States) to cross the aortic arch. The sheath was exchanged with a 7-Fr 90-cm-long sheath (Arrow International, Brooklyn, Ohio, United States). The HAAs were catheterized with a 5-Fr Bern catheter 100 cm (Boston Scientific). An angiography was performed which revealed the presence of small collaterals in the liver hilum and the absence of right and left hepatic arteries (Figure 3).

**Figure 4:**
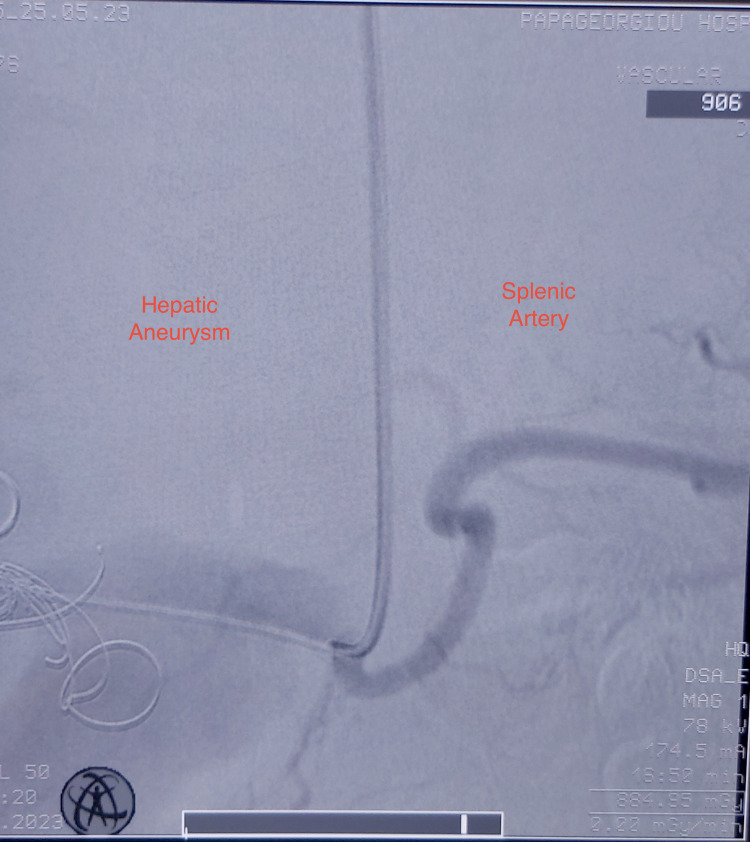
Intraoperative angiography showed the bifurcation of the hepatic and splenic artery with inadequate proximal landing zone.

Seven coils (Interlock, Boston Scientific) of different sizes 18×20, 18×40, and 20×40 were released to the distal part of the aneurysm. Because of the lack of a proximal landing zone as well as the poor liver perfusion from the hepatic artery, we decided to exclude the inflow of the aneurysm by deploying a balloon-expandable covered stent 9×37 mm (Bentley®, Innomed, Germany) from the celiac artery to the splenic artery (Figure 4).

**Figure 5:**
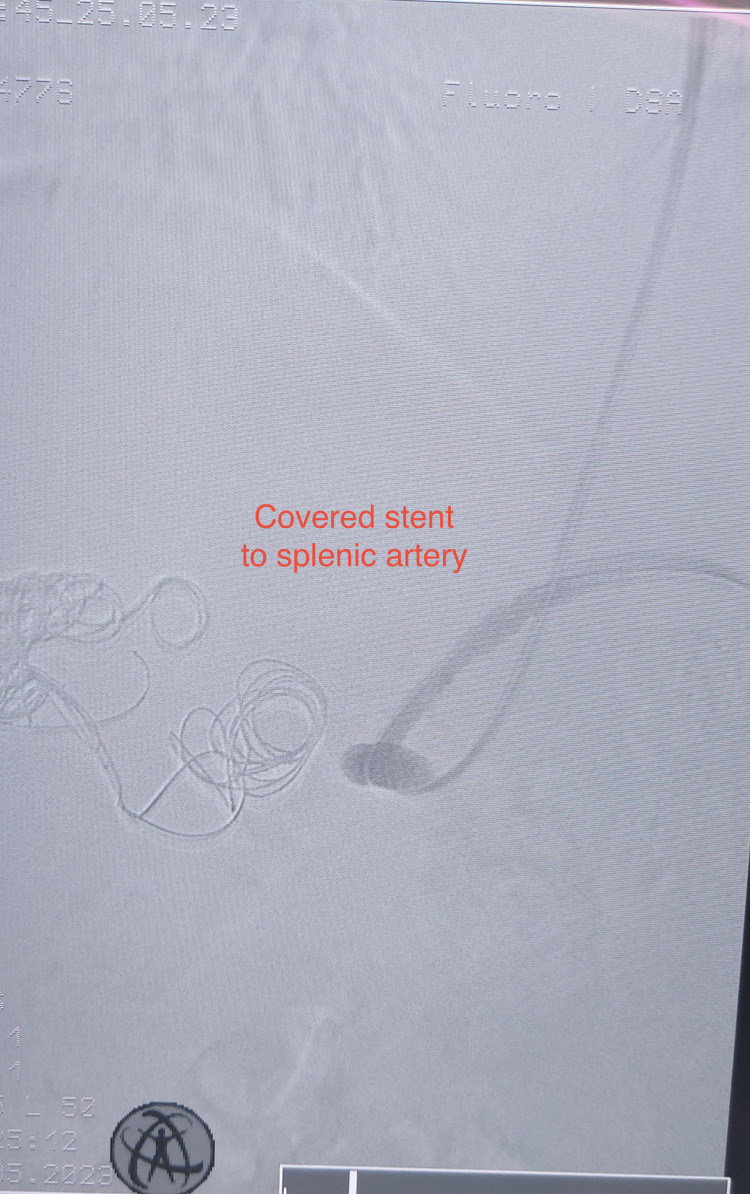
Deployment of the covered stent to the splenic artery with inflow exclusion of the hepatic aneurysm.

The final angiographic control revealed the occlusion of the aneurysm and perfusion of the splenic artery (Figure 5). 

**Figure 6:**
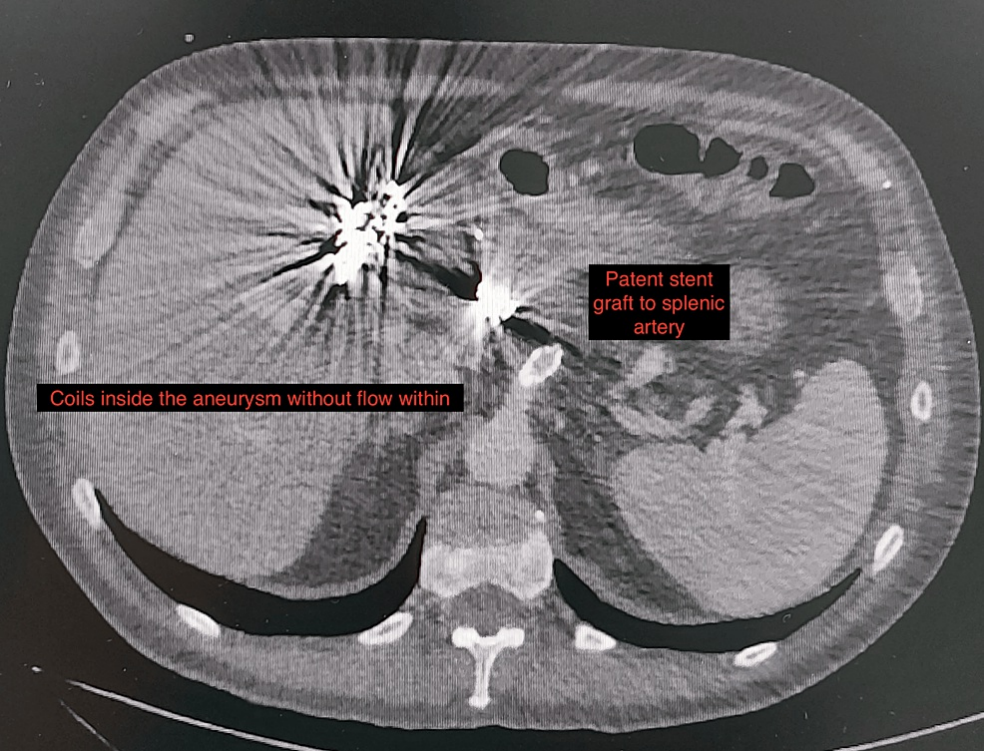
CTA at six months showed shrinkage of the aneurysm with no flow within and patent stent to the splenic artery. CTA: computed tomography angiography

The perioperative period was uneventful without any clinical and laboratory signs of liver ischemia. The patient was discharged on the fourth postoperative day (POD) under dual antiplatelet treatment (Salospir 100+clopidogrel 75 mg) for three months followed by a single lifelong antiplatelet treatment and proton pump inhibitor regimen. No significant changes in the hepatic enzyme's levels were observed during follow-up. At the three-month follow-up, the patient was free of symptoms, and the computed tomography angiography (CTA) showed a significant reduction in the size of the aneurysm. CT scan at six months revealed excellent patency of the stent graft to the splenic artery and shrinkage of hepatic aneurysm at 5 cm (Figure 6).

**Figure 7:**
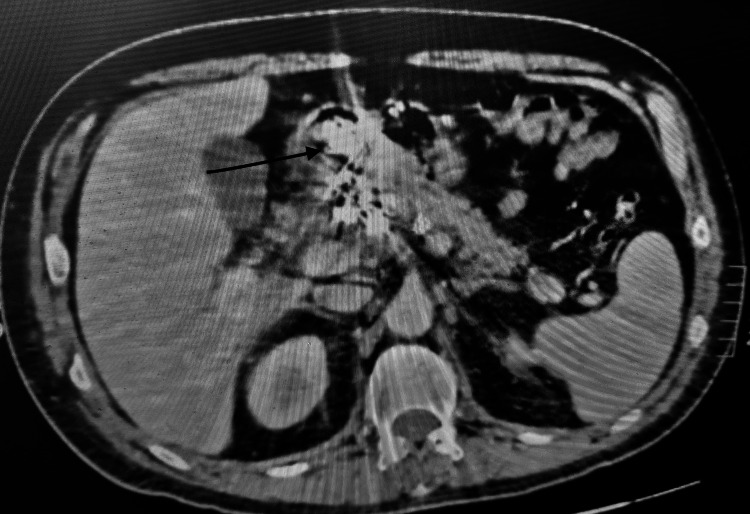
Contrast-enhanced CT axial view depicted the pyloric erosion and protrusion of coils in the proximal part of the duodenum (black arrow). CT: computed tomography

**Figure 8:**
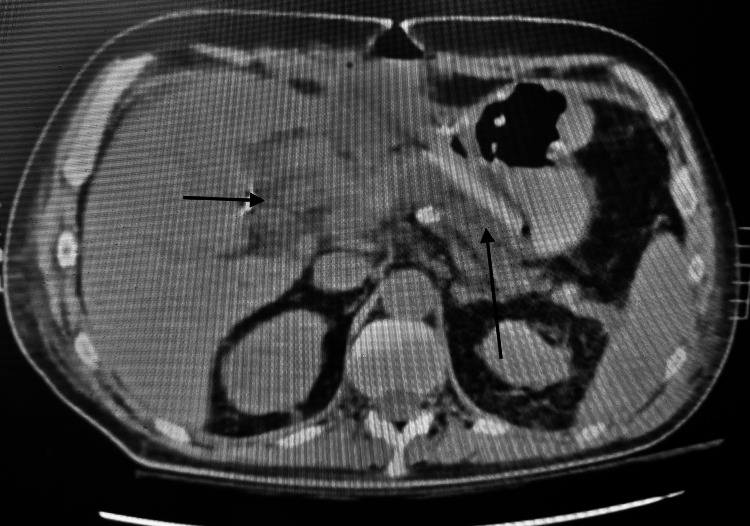
CT view after the removal of coils within the aneurysm (small arrow) and placement of drainage tube next to the lesion (long arrow). CT: computed tomography

However, seven months after the operation, the patient presented to another hospital with upper gastrointestinal hemorrhage and anemia. A contrast-enhanced CT (Figure 7) and gastroduodenoscopy were performed which revealed a pylorus perforation with the presence of coils entering the lumen. A laparotomy was performed. After gastrotomy, multiple coils were found in the duodenum, as well as an arterio-pyloric fistula. Coils were removed, and a Heineke-Mikulicz pyloroplasty was performed with the placement of drainage next to the perforation site (Figure 8). His postoperative course was uncomplicated, and the patient was discharged on the 12th POD. 

## Discussion

HAAs, especially giants, are a rare pathological entity which, if left untreated, result in catastrophic consequences. Historically, open repair with aneurysmectomy and interposition graft placement was the treatment of choice. Nowadays, endovascular approach as a minimally invasive therapy offers lower morbidity and mortality and less duration of hospitalization [5]. Different endovascular techniques are available (packing embolization, isolation embolization, stent graft deployment) depending on the aneurysm's anatomy [6]. A narrative literature review regarding the endovascular repair of giant HAAs was performed. The search was conducted by two authors (KS, KT) for records published up until January 2024 and using the term "hepatic artery aneurysm" in the PubMed, Scopus, and Google Scholar databases (Figure 5). 

**Figure 9:**
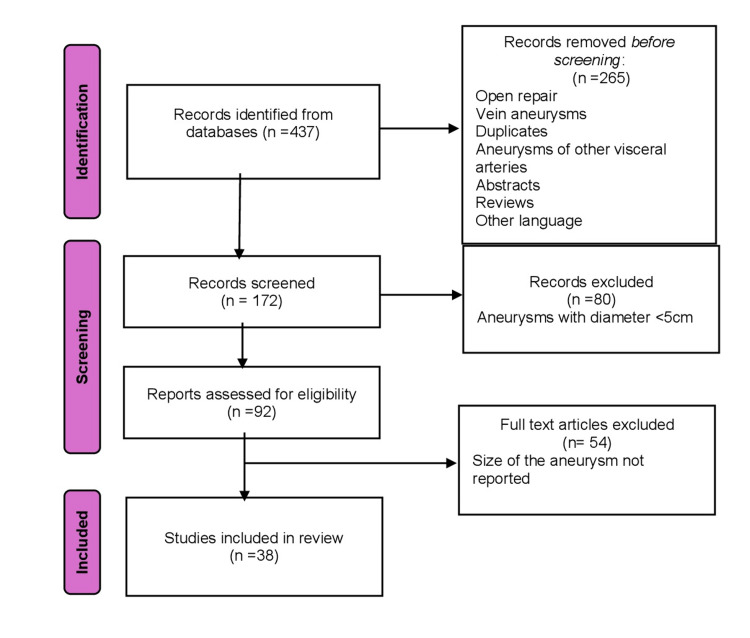
Flowchart diagram.

Screening was performed by title and abstract and in some cases using the full-text article. Eligibility criteria were HAAs with a minimum diameter of 5 cm that were treated using an endovascular approach. Manuscripts written in the English language were included in the review. Thirty-eight studies met the eligibility criteria [7-45] (Table 1). Male is the prevalent gender (73.2%). The mean age is 60.63 years, while in one case age is not reported. In 16 cases, the size of the aneurysm exceeds 8 cm in diameter (39%). The most common location of the aneurysm is the common hepatic artery (73.2%), proper hepatic artery (12.2%), right hepatic artery (9.7%), and left hepatic artery (4.9%). Arterial access was obtained in the majority of the cases through the femoral artery (58.5%), followed by the left brachial artery (9.7%), while in 13 cases (31.7%), vascular access was not reported.

**Table 1:** List of published studies regarding the endovascular repair of giant hepatic artery aneurysms. NM: not mentioned

Author and year	Sex	Age	Location/type	Size (cm)	Access site	Endovascular intervention	Complication
Larson et al. (2002) [8]	Male	73	Common hepatic artery/pseudoaneurysm	8	Femoral artery	Stent graft	None
Qu et al. (2005) [9]	Male	72	Common hepatic artery/pseudoaneurysm	10.3	Left femoral artery	Endoaortic stent graft complex	None
Dwivedi et al. (2006) [10]	Male	56	Common hepatic artery/NM	8	Left femoral artery	Coils	None
Jenssen et al. (2007) [11]	Male	61	Common hepatic artery aneurysm/true aneurysm	6	Right and left femoral artery	Stent graft	None
deFreitas et al. (2007) [12]	Female	71	Common hepatic artery aneurysm/pseudoaneurysm	5.2	Brachial artery	Stent graft	None
Desai and Sekhar (2008) [13]	Male	57	Common hepatic artery aneurysm/pseudoaneurysm	8.9	ΝΜ	Coils	None
Won et al. (2009) [14]	Male	72	Common hepatic artery aneurysm/pseudoaneurysm	5	Femoral artery	Stent graft	None
Miraglia et al. (2010) [15]	Male	52	Left hepatic artery/pseudoaneurysm	5	NM	Coils, vascular plug	None
Balderi et al. (2010) [16]	Male	74	Common and proper hepatic artery aneurysm/NM	8.5	Right femoral artery	Multilayer stent	None
Christie et al. (2011) [17]	Male	69	Common hepatic artery/pseudoaneurysm	11	NM	Coils	None
Huisman et al. (2011) [18]	Female	48	Proper hepatic artery aneurysm/true aneurysm	6.3	Right femoral artery	Bare metal stent, fibrin glue	None
Ferrero et al. (2013) [19]	Male	71	Common hepatic artery/NM	6	ΝΜ	Stent grafts	In 18 months, stent dislocation and sac expansion. Eventually, stent and CHA thrombosis
Cavalcante et al. (2014) [20]	Female	56	Common hepatic artery and splenic artery/saccular aneurysm	12.5	Right femoral artery	Vascular plug	None
Buyukkaya et al. (2014) [21]	Female	49	Proper hepatic artery/pseudoaneurysm	9.1	ΝΜ	Covered stent	None
Rossi et al. (2015) [22]	Male	69	Common hepatic artery/NM	10	NM	Non-adhesive liquid embolic ethylene-vinyl alcohol agent (Onyx®), vascular plug	None
Hemmati et al. (2015) [23]	Female	67	Common hepatic artery/true aneurysm	9.5	Left brachial artery	Coils	None
Yoon et al. (2016) [24]	Male	67	Common hepatic artery/true aneurysm	15	Right femoral artery	Stent grafts, metallic stents	None
Pedersoli et al. (2016) [25]	Male	56	Common hepatic artery/pseudoaneurysm	6.5	Right femoral artery	Stent graft	None
Attaoui et al. (2016) [26]	Male	80	Proper hepatic artery/NM	6.2	NM	Coils	None
Zhang and Li (2016) [27]	Female	7	Right hepatic artery/pseudoaneurysm	5.8	ΝΜ	Coils	None
Abdallah et al. (2017) [28]	Male	66	Common hepatic artery/true aneurysm	11	NM	Coils, non-adhesive liquid embolic ethylene-vinyl alcohol agent	None
Abdelbaki et al. (2017) [29]	Male	82	Right hepatic artery/pseudoaneurysm	14	ΝΜ	Coils	None
Soares et al. (2017) [30]	Male	65	Common hepatic artery/true aneurysm	7.5	Right femoral artery	Covered stent	None
Ferrara et al. (2017) [31]	Male	79	Common hepatic artery aneurysm/true aneurysm	7.2	Right femoral artery and left brachial artery	Stent grafts	None
Akasaka et al. (2018) [32]	Female	73	Proper hepatic artery aneurysm/true aneurysm	6.5	Right femoral artery	Microcoils	None
Masuda et al. (2019) [33]	Female	68	Common hepatic artery/true aneurysm	9	Right and left femoral artery	Coils, vascular plug	None
Gjoreski et al. (2019) [34]	Female	NR	Common hepatic artery/true aneurysm	7	Brachial artery	Closed-cell and double-layer micromesh stent	None
Ferreras et al. (2019) [35]	Male	45	Common hepatic artery and proper hepatic artery/pseudoaneurysm	6	NM	Hydrocoils, vascular plug	Immediate hepatic failure. The patient underwent liver transplantation
O'Connell et al. (2020) [36]	Male	77	Common hepatic artery/true aneurysm	5.2	NM	Covered stents	None
Kwon et al. (2020) [37]	Male	61	Common hepatic artery/true aneurysm	6	Right femoral artery	Coils	None
Hashem and Peddu (2020) [38]	Female	54	Common hepatic artery/true aneurysm	7.6	Right femoral artery	Covered stents in the common hepatic artery, coils in the gastroduodenal artery	None
Gao et al. (2021) [39]	Male	69	Common hepatic artery/true aneurysm	5.3	Right femoral artery	Covered stents in the common hepatic artery, coils in the gastroduodenal artery	Three months: sepsis, aerobilia, in-stent thrombosis, endoleak Ia. The patient underwent open repair
Mahmood et al. (2021) [40]	Male	35	Left hepatic artery aneurysm/pseudoaneurysm	5.8	NM	Gel foam, microcoils	None
Kar and Patel (2021) [41]	Male	50	Common hepatic artery/pseudoaneurysm	5.3	Right femoral artery	Coils, thrombin injection, stent graft	(1) In six months, endoleak: coils and stent graft. (2) In six years, coil extrusion into the duodenum and arterioenteric fistula: open repair
Tipaldi et al. (2021) [42]	Male	69	Hepatic artery/true aneurysm	10	Right femoral artery	Vascular plug, non-adhesive liquid embolic ethylene-vinyl alcohol agent (Onyx®)	None
	Male	49	Hepatic artery/true aneurysm	5.6	Right femoral artery	Peripheral occlusion device	None
	Male	63	Hepatic artery/true aneurysm	5.2	Right femoral artery	Covered stent	None
	Male	69	Hepatic artery/true aneurysm	13	Right femoral artery	Coils, vascular plug, peripheral occlusion device	None
Clark et al. (2023) [43]	Male	44	Proper hepatic artery/true aneurysm	5.5	Brachial artery	Covered stents	Three weeks: abdominal pain and endoleak via collateralization, followed by coil embolization
Çildağ et al. (2023) [44]	Female	60	Right hepatic artery/true aneurysm	5	Right femoral artery and brachial artery	Coils	None
Ghasemi-Rad et al. (2023) [45]	Male	20	Right hepatic artery/pseudoaneurysm	6.5	Femoral artery	Balloon-assisted thrombin injection	None

Fifteen patients (36.6%) were treated using stent grafts and closed-cell and double-layer micromesh stent [8,11,12,14,16,19,21,24,25,30,31,34,36,42,43]. Ten patients were treated exclusively by coils (24.4%) [10,13,17,23,26,27,29,32,37,44], but in most cases, a combination of endovascular methods was used (liquid embolic agent+vascular plug [22,42], coils+vascular plug [15,33,35], covered stents+coils [38,39], coils+liquid embolic agent [28,40], coils+vascular plug+peripheral occlusion device [42]). In one case, a stent graft complex was deployed into the aorta to seal the orifice of the celiac trunk, due to aneurysm extension and lack of proximal landing zone [9].

Postoperative complications were reported in five cases (13.5%) [19,35,39,41,43], whereas the mortality rate was 0%. Two of the five cases were managed with an endovascular approach [19,43], while the rest with an open repair [35,39,41]. Ferrero et al. reported that a 3.4 cm HAA previously treated with two multilayer stents showed a stent dislocation and sac expansion in 18 months follow-up [19]. The patient was treated with a new multilayer stent placement, but due to incorrect stent expansion, all three stents were thrombosed. However, the perfusion of the proper hepatic artery remained adequate, due to rich collateralization. In Ferreras et al., the patient experienced a fulminant hepatic failure after the embolization and was subjected to an immediate liver transplantation [35]. In Clark et al., a coil embolization within the sac and the covered stent was required three weeks postoperatively due to endoleak through collaterals [43]. In Gao et al., an open repair was chosen due to presented aerobilia, sepsis, in-stent thrombosis, endoleak Ia, and infection of the aneurysm [39]. Lastly, Kar and Patel report a recurrent filling of the aneurysmal sac in six months, which was treated with coil, stent graft, and plug placement [41]. After six years, the patient presented massive gastrointestinal bleeding, and the CTA showed a coil extrusion into the duodenum in the form of an arterioenteric fistula; hence, the patient underwent open repair. A duodenotomy was performed, the coils, the stent graft, and the plug were removed, and the celiac artery trunk along with the left gastric artery was ligated.

It is interesting that only one patient with giant HAA experienced acute ischemic hepatic failure after the embolization technique of distal outflow [35]. The liver has a dual blood supply from the portal vein (70%) and the hepatic artery (30%). Approximately 50% of oxygen demand is met by the portal vein and 50% by the hepatic artery [46]. Interruption of hepatic collateral circulation distal to the origin of the gastroduodenal artery can impair hepatic perfusion. However, the buffer response of the portal venous system with the ongoing angiogenesis which has been described during aneurysm evolution and is related to the presence of collateral circulation can minimize ischemia [46,47]. In our case, the patient had a patent portal venous system with the presence of small hepatic arterial collaterals in the liver hilum which probably minimized hepatic ischemia.

Endovascular treatment of giant visceral aneurysms offers different techniques such as coils, plugs, covered stents, glue, and microcatheters which can exclude flow in the aneurysm. Anatomical characteristics of the aneurysms like length, angulation, tortuosity, and proximal and distal landing zones should be evaluated preoperatively to choose the appropriate endovascular modality. In our case, we chose the double inflow-outflow blockage of the aneurysm by two endovascular means, proximally covered stent to deviate the flow to the splenic artery since there was inadequate proximal landing zone and distally coils to prevent backflow to the aneurysm. However, the patient presented seven months later with pylorus erosion due to coils and upper gastrointestinal hemorrhage and was managed by open repair. Kar and Patel [41] described duodenal erosion by coils six years after the operation. They concluded that the number of coils used to thrombose the aneurysm should be at the minimum to achieve hemostasis. 

## Conclusions

Endovascular repair of giant hepatic aneurysms is an effective and feasible therapeutic approach. Proper preoperative planning and correct selection of endovascular devices are mandatory to achieve the exclusion of the aneurysm. A great concern should be raised regarding the possible erosion of the upper gastrointestinal tract by coils used for embolization during follow-up.
